# Rehabilitation of a Midfacial Defect Using a Two-Piece Maxillofacial Prosthesis: A Case Report

**DOI:** 10.7759/cureus.22138

**Published:** 2022-02-11

**Authors:** Chakradhar Vadlamudi, Lakshmana Rao Bathala, Satyanarayana S V Tammineedi, Bhargavi Bandlamudi, Parvathi PSHL

**Affiliations:** 1 Department of Prosthodontics, Lenora Institute Of Dental Science, Rajahmundry, IND

**Keywords:** neoplasms, speech, maxillofacial prosthesis, mastication, zygoma, midfacial defects

## Abstract

Maxillofacial defects and their rehabilitation are a major concern in this socially productive era. The rehabilitation of these massive defects in the oral and maxillofacial region poses a challenge to the prosthodontist in terms of selection of material, retentive aids, the adaptive capability of the patient, and cost. This case report describes the management of the midfacial defect involving the orbit, zygoma, maxilla, and their soft tissue counterparts with a removable silicone extraoral compartment and an acrylic intraoral compartment, which are retained with strong cobalt samarium magnets, an elastic loop around the occiput, and spectacles. The maxillofacial prosthesis fabricated for this patient restored the patient’s facial esthetics, speech, dental articulation, lip support, mastication, and anterior maxillary seal.

## Introduction

The main etiology of maxillofacial defects is either congenital or acquired. The acquired defects may be caused by different pathologies, radiation burns, trauma, and surgical interventions. Marunick et al. classified midfacial defects into (1) midline defects, including the nose and upper lip, and (2) lateral defects, which include the cheek and orbital portions. Additionally, combinations of these categories can be found [1].

The main goals of prosthetic rehabilitation of maxillofacial defects are to restore the patient’s facial esthetics and function of the associated structures and to maintain the integrity of the remaining tissues. These maxillofacial defects are difficult to restore with a prosthesis because of a lack of bony support, scar tissue formation, and their enormous size [2]. Alternative retention strategies include eyeglasses, elastic bands, magnets, adhesives, combinations of the above, and implants [3-7].

The patient treated in this report was previously rehabilitated with a facial prosthesis [8], the main drawbacks of which were color instability and loss of marginal integrity, for which complete refabrication using a two-piece prosthesis was planned. This case report describes the fabrication of a two-piece prosthesis, for which an intraoral prosthesis refabrication was not required. Additionally, an extraoral prosthesis can be refabricated every six months to one year based on the properties of the silicone. This case report differs from previous reports in terms of fabrication technique, iris positioning, and type of auxiliary retention system used. Because the soft tissues had incomplete healing and there was minimal osseous tissue, we selected a removable prosthesis as a treatment option for this clinical situation.

## Case presentation

**Figure 1:**
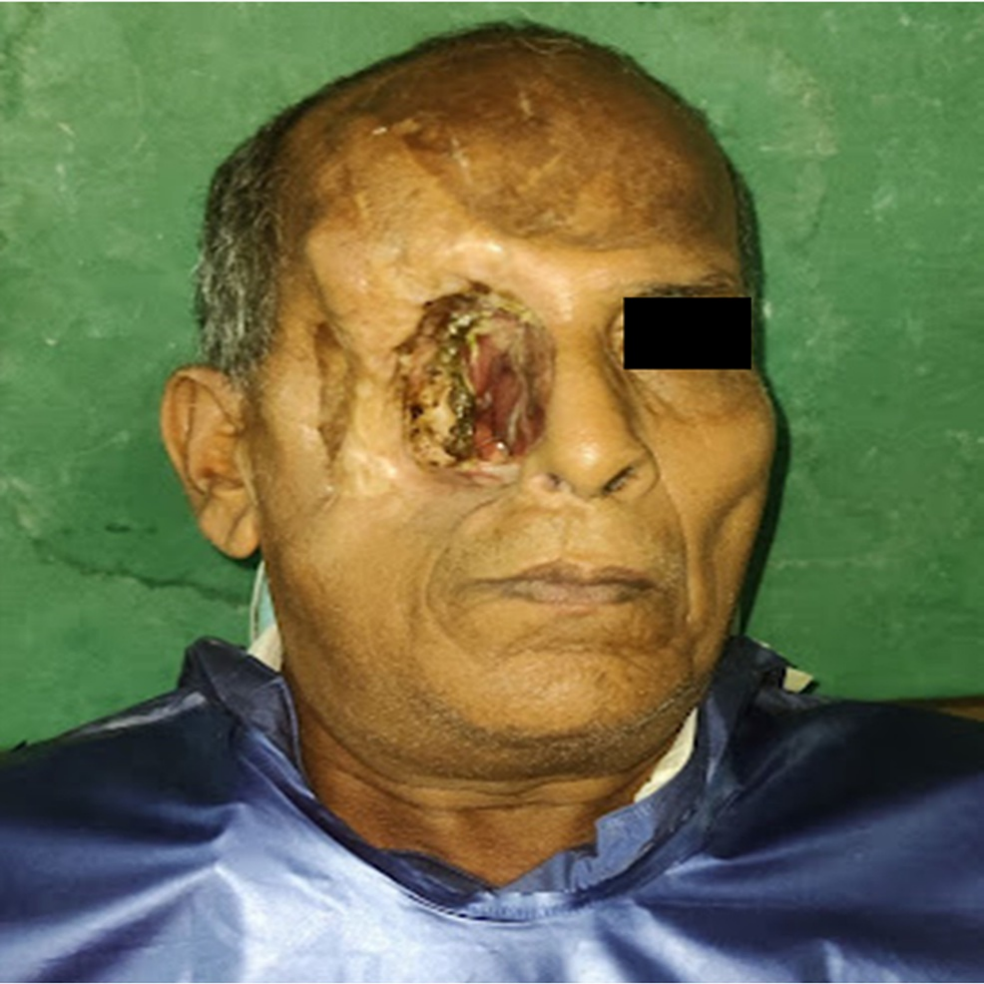
Preoperative image of the patient revealing a lost zygomatic arch and its soft tissues and orbital contents on the right side of the face

A 56-year-old male patient was referred to the Department of Prosthodontics, Lenora Institute of Dental Sciences, Rajahmundry, India, for maxillofacial rehabilitation. Past medical history includes that the patient was known to be diabetic and was under medication and had a history of surgical resection of the right side's hard palate, zygomatic arch, and orbital contents due to mucoepidermoid carcinoma (Figure 1). The patient was previously treated with the obturator that was not retentive enough to hold the massive intraoral and extraoral compartments. Intraoral examination revealed partial maxillary defect and teeth from the lateral incisor to the second molar on the right side of the arch, communicating with the extraoral defect. There is generalized attrition and staining on the maxillary and mandibular teeth. Tongue movements were normal without obstruction. Temporomandibular movements were normal without any deflection or deviation.

**Figure 2:**
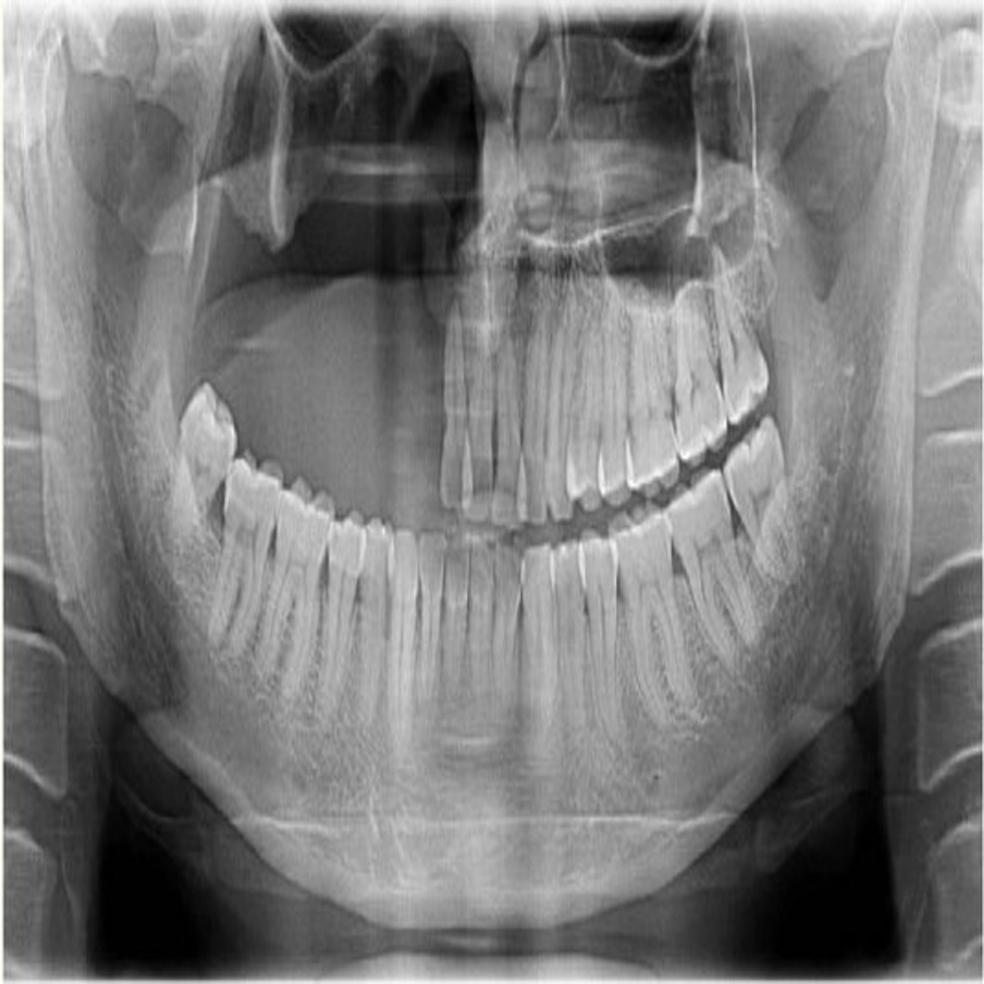
Orthopantomagram revealing the residual bone and intraoral status

Extraoral findings include the following: 1. loss of the zygomatic arch, orbit, and soft tissue contents; 2. the presence of redness, inflammation, and debris in the unhealed soft tissues surrounding the defect; 3. tissue scarring and mismatched areas as a result of the tissue grafting procedure. The intraoral findings were confirmed and re-evaluated with the orthopantomogram (OPG) radiograph (Figure 2). The OPG revealed thin cancellous bone around the resected zygomatic, pterygoid, and orbital residues, which makes it unfavorable for the placement of maxillofacial implants. The residual bone type, amount, contours, and status of the remaining teeth were assessed, and a treatment plan was planned accordingly.

The patient had a chief complaint of difficulty with speech, mastication, and swallowing. He was facing social difficulties when people questioned him about the cause of his deformity and treated him as if he was disabled.

After reviewing all the constraints of this case, the proposed treatment plan was to fabricate a removable prosthesis with magnetic attachment to an acrylic obturator and a silicone facial prosthesis. The retentive aids used were cobalt-samarium magnets, spectacles with elastic head loops, and adhesives. The treatment protocol follows the sequence of making an impression-pouring working model, followed by wax pattern fabrication, sculpting, try-in and iris positioning, packing and curing with silicone, prosthesis try-in, and insertion after adequate modifications.

Creation of the impression

**Figure 3:**
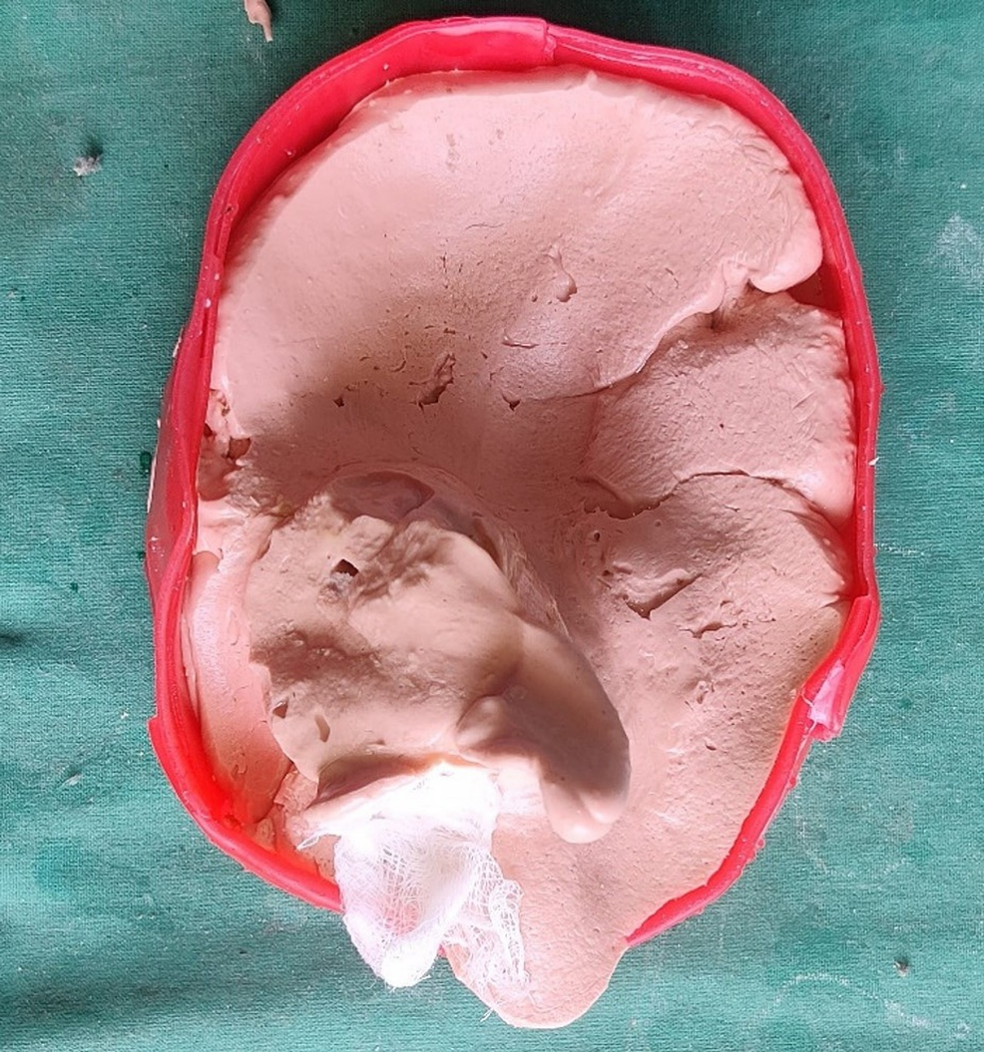
Impression of the facial moulage of the patient using irreversible hydrocolloid

Facial moulage was made using an irreversible hydrocolloid impression material (Algitex, Dental Products of India, Mumbai, India). To prevent the impression material from flowing into the anatomical undercut areas, separation media were applied before making an impression. After the impression was made, it was boxed to confine the borders and poured using Hydrocal (gypsum product) (Figure 3).

Wax pattern try-in and positioning of the iris

**Figure 4:**
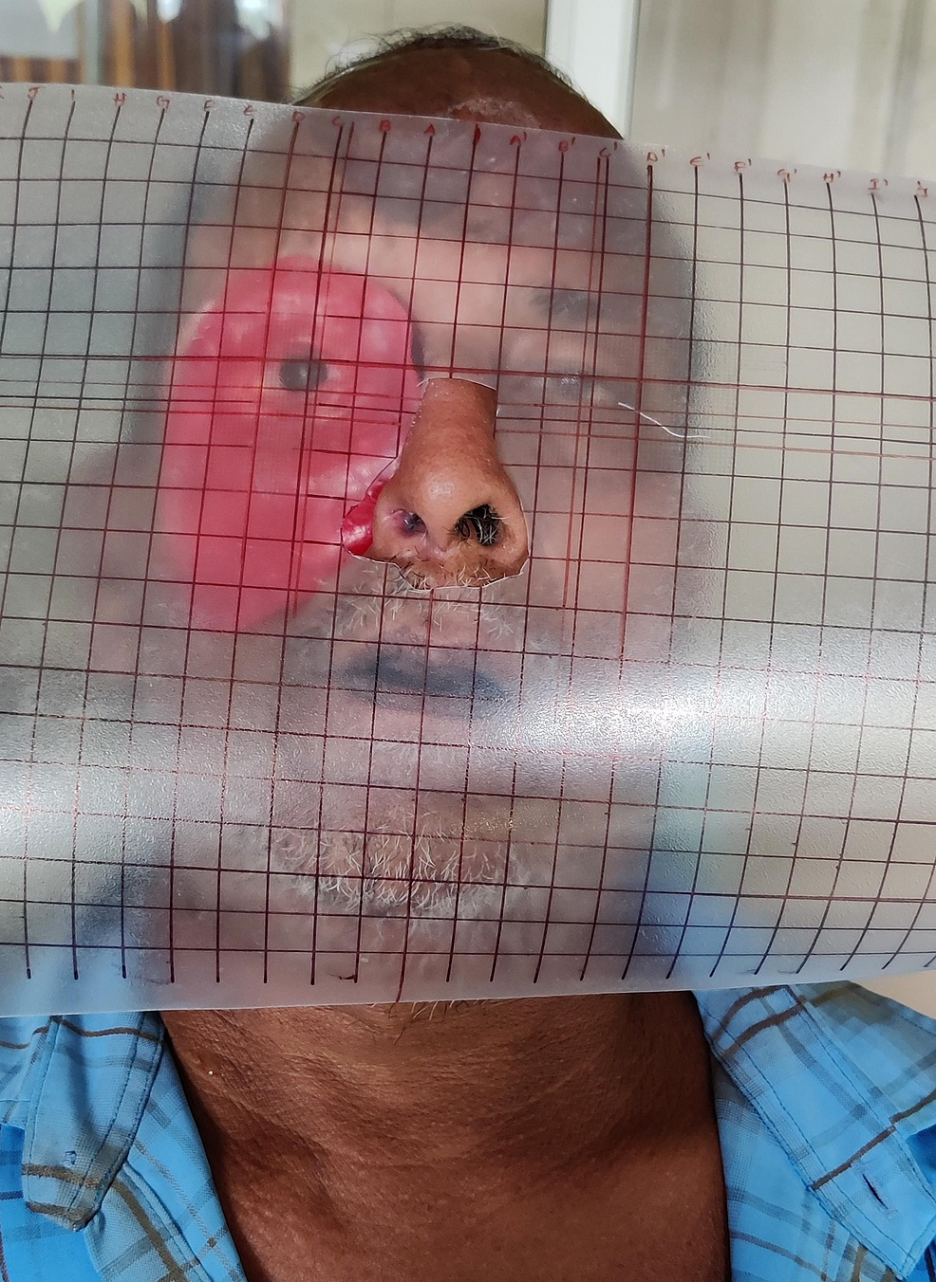
Wax pattern try-in and iris positioning for the prosthesis

The wax pattern was created. Subsequently, the iris of the stock eye was positioned by taking its counterpart as a reference (Figure 4).

Packing and curing

After dewaxing, the available mold space was packed with vulcanizing silicone at room temperature. Using intrinsic pigments, intrinsic staining was done to match the patient's hue (Factor II, Inc., Lakeside, AZ). This curing process was performed following the manufacturer’s recommendations. The cured prosthesis was retrieved and finished.

Prosthesis try-in

**Figure 5:**
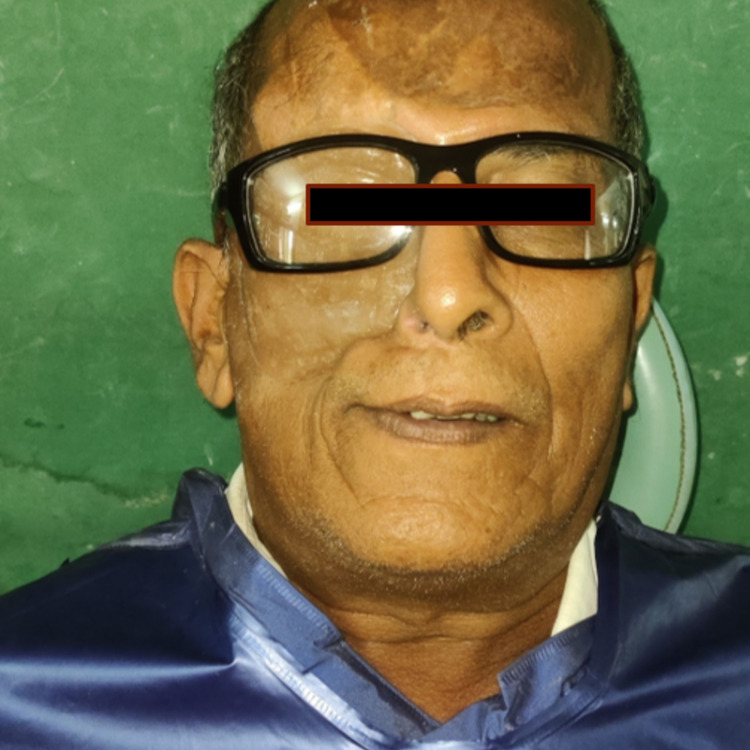
Extraoral try-in and insertion of the silicone prosthesis retained with spectacles and an elastic head loop

The facial prosthesis was tried in the defect area, and a cobalt-samarium magnet of 10-mm diameter, adhesives, and spectacles with an elastic head loop (thick borders were selected to mask the margins of the prosthesis) were selected as auxiliary retentive aids (Figure 5).

Intraoral prosthesis

**Figure 6:**
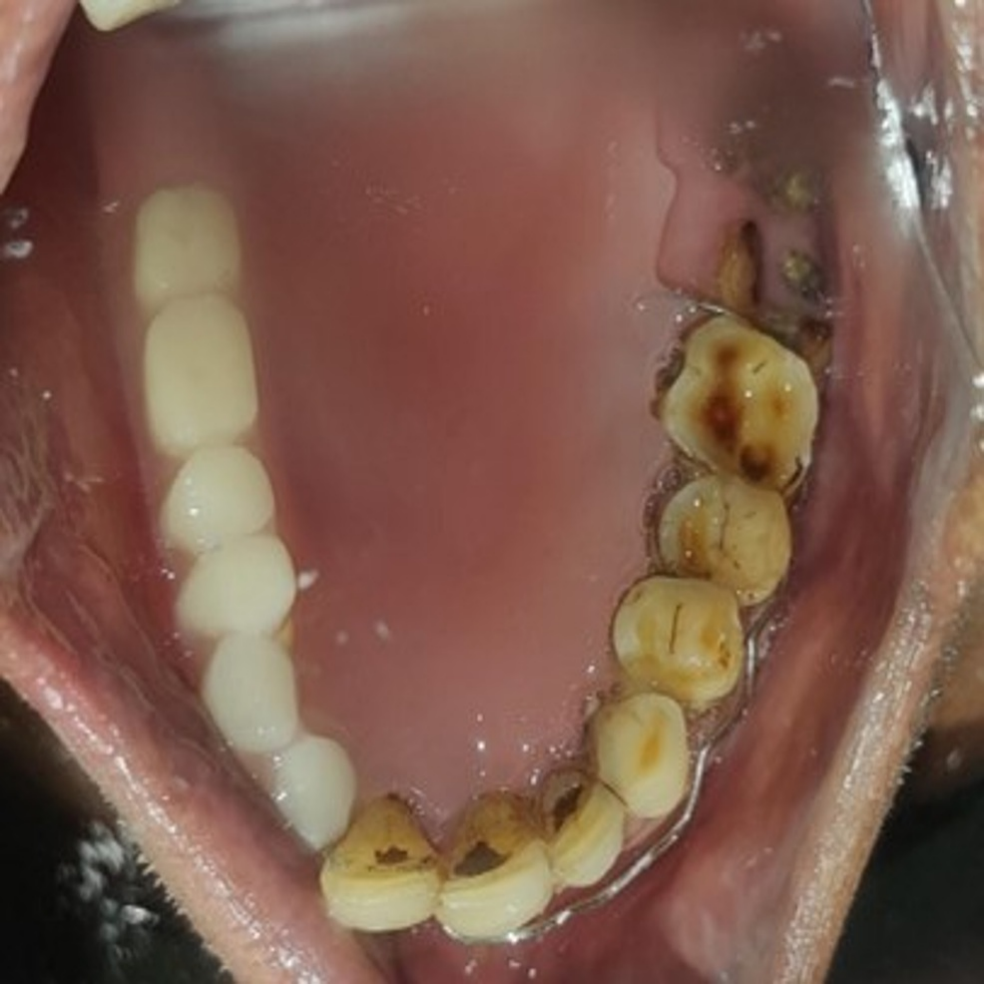
Intraoral insertion of the prosthesis

**Figure 7:**
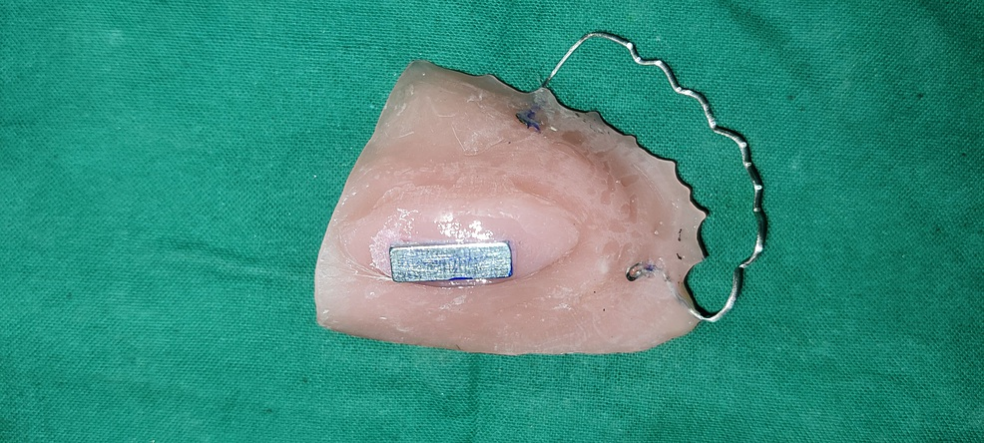
Intraoral prosthesis with a rectangular cobalt–samarium magnet and clasp

The patient had a missing tooth in relation to numbers 12, 13, 14, 15, 16, and 17 in the continuation of the extraoral defect. The intraoral prosthesis was designed in the same way as a conventional acrylic partial denture but by involving the defect. We used a clasp and cobalt-samarium magnet of 2.0-mm thickness (rectangular shaped) to help in retaining the intraoral prosthesis (Figures 6-7).

Follow-up

Post-insertion instructions were given and recalled after one week, one month, three months, and six months. During this period, post-insertion problems mainly were with the prosthesis and retention of the two components. The prosthesis was relined intraorally, and the retention was improved by changing the path of placement of the prosthesis. The patient’s satisfaction with the treatment, comfort with the prosthesis, and retention between the two compartments of the prosthesis were assessed with a visual analog scale during these recall visits. The patient's satisfaction was improved from score 6 to score 9 gradually during the visits; after the final appointment, the patient was satisfied with the comfort and esthetics of the prosthesis.

## Discussion

**Figure 8:**
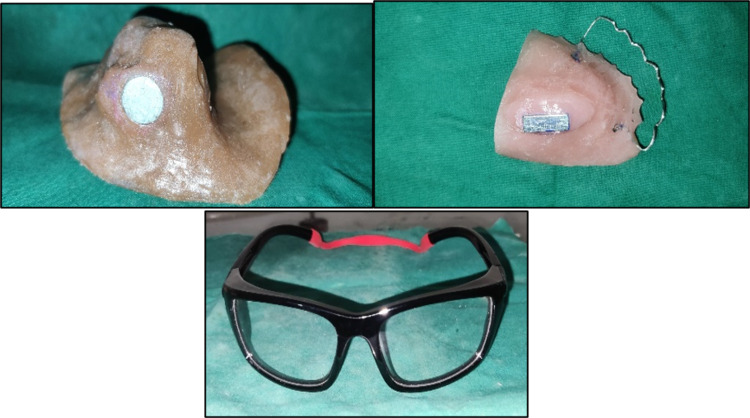
The retentive aids used includes cobalt samarium magnets and spectacles with an elastic head loop

Prosthetic rehabilitation has become an alternative, economical, non-invasive option in large defect reconstruction where the plastic surgical approach [9] poses many technical, biological, and anatomical difficulties. The patient reported in this article lost his right maxilla, zygoma-associated structures, and orbital contents due to resection of mucoepidermoid carcinoma. This type of midfacial defect can be managed by using zygomatic and pterygoid implants [10-11], combination prostheses [5], magnets, adhesives, elastics, spectacles, magnets, key-lock mechanisms, and the combinations of many other auxiliary retentive aids. According to Gaur V et al. using one-piece implants with bicortical anchorage could be an additional tool in maxillofacial defect reconstruction [5]. Vega LG et al. suggested that using zygoma implants to reconstruct an acquired maxillary defect is a safe, predictable, and cost-effective treatment option [12]. Advancement in the progress of implants suggested that the reverse zygomatic implant appears to hold promise as a useful addition to the implant arsenal for the treatment of maxillectomy patients [13]. Literature suggests that the atrophic bone contents induce no osseointegration with zygomatic implants, leading to periimplantitis and complications [14-15]. Some of the surgical placement complications of the pterygoid implants reported in the literature include minor venous bleeding, minor trismus, implant displacement, and a unique case of continuous pain and discomfort [16-18]. The main reasons for the elimination of the implant placement are the surgical post-surgical complications and the present psychological status of the patient who was refrained from the surgical treatment option and the cost factor. However, the sectional or removable prosthesis has several advantages while removing the prosthesis and in the maintenance phase [5]. So, the rehabilitative procedure planned should be non-invasive and cost-effective, and deliver the aesthetics and functional requirements of the patient. So, the room temperature medical-grade silicone, acrylic, cobalt samarium intraoral magnets, elastic loop encircling the occiput, and spectacles were selected and used efficiently to fabricate the combination prosthesis. The patient’s previous prosthesis was retained using small iron-cobalt magnets [8]. These were replaced with cobalt samarium, which has strong magnetism [6]. A 10 mm diameter extraoral silicone prosthesis was used. A cylindrical magnet of 8 4 mm on the tissue side of the intraoral acrylic prosthesis was used to face the unlike poles to have strong magnetism. Extraoral retention and adaptability were executed with the help of head loops and spectacles (Figure 8). The combination of intraoral and extraoral retentive aids in the proper positioning of the prosthesis.

Reversible hydrocolloid (Alginate) was used instead of elastomeric impression materials because of the flow of the hydrocolloid with minimal force application and easy removal if engaged in anatomical undercuts. Medical grade silicone and silicone adhesives were used in this case to maintain biological compatibility with the tissues. As concluded from the study of Dos Santos DM et al. [19]. Intrinsic staining was used to increase the life-like appearance of the prosthesis decrease the fading of the color of the silicone prosthesis. However, extrinsic staining was done in his previous prosthesis, which later faded rapidly within six months. The main limitations of the silicone prosthesis are its weight and the difficulty of hollowing it out and retaining the prosthesis. In this report, mechanical retentive aids, such as magnets in the facial prosthesis and intaglio surfaces of the removable prosthesis with unlike poles of the magnets facing each other, were used to overcome the retention problem. The advantage of the current study was that the fading silicone prosthesis could be exchanged periodically without replacing the intraoral prosthesis; the weight of the prosthesis was reduced, as it was made into two pieces. The retentive aids used and the procedure followed were economical and less technique-sensitive.

## Conclusions

The two-piece prosthesis helps in the retention of each other with the help of magnets and distributes the forces between the prostheses. If needed, a silicone prosthesis can be refabricated without the fabrication of an intraoral acrylic prosthesis. The technique is non-invasive, economical, esthetically acceptable, comfortable, maintainable, and atraumatic.

The rehabilitation of the defects in the oral and maxillofacial region needs a multidisciplinary approach. The terminal specialist to encounter during this process was a prosthodontist. Therefore, it is the prosthodontists' duty to plan the treatment according to the clinical condition and explain the odds in the treatment process.
